# Upregulation of FBXW7 Suppresses Renal Cancer Metastasis and Epithelial Mesenchymal Transition

**DOI:** 10.1155/2017/8276939

**Published:** 2017-09-30

**Authors:** Yangke Cai, Meng Zhang, Xiaofu Qiu, Bingwei Wang, Yu Fu, Jun Zeng, Jian Bai, Guosheng Yang

**Affiliations:** ^1^Department of Urology, Guangdong Second Provincial General Hospital, The Third Clinical Medical College of Southern Medical University, Guangzhou, Guangdong, China; ^2^Department of Urology, The First Affiliated Hospital of Anhui Medical University, Hefei, China

## Abstract

**Background and Objective:**

FBXW7, known as a general tumor suppressor, is commonly lowly expressed in metastatic malignancies. We aim to investigate the potential influence of FBXW7 overexpression on renal cell carcinoma (RCC) metastasis.

**Methods:**

We employed quantitative real-time PCR (qRT-PCR) and Western blotting (WB) to quantify the FBXW7 expression in RCC cell lines. Upregulation of FBXW7 was performed *in vitro* on RCC cells using the lentivirus covering coding region FBXW7 cDNA sequence, and functional tests were performed to verify FBXW7 overexpression on migration and invasion of RCC cells. Moreover, WB was employed to determine the expressions of MMP-2, MMP-9, and MMP-13, as well as EMT markers in the transfected RCC cells.

**Results:**

FBXW7 was significantly downregulated in RCC cell lines, dominated by 786-O and ACHN, when compared to normal renal cell line HK-2. Moreover, upregulation of FBXW7 in 786-O and ACHN cell lines significantly inhibited cell migration and invasion, as well as EMT. Present study also showed that FBXW7 was involved in the migration and invasion of RCC cells via regulating the expressions of MMP-2, MMP-9, and MMP-13.

**Conclusion:**

Our findings demonstrate that upregulation of FBXW7 inhibits RCC metastasis and EMT. FBXW7 is a potential therapeutic target for RCC patients.

## 1. Introduction

Renal cell carcinoma (RCC) on behalf of a heterogeneous group of tumors originates from the kidney. Diverse tumor types falling under the umbrella of RCC comprise clear cell, papillary, and chromophobe, which represent 65%, 20%, and 5% of all RCC cases, individually [1]. Surgical excision with total nephrectomy remains to be the standard curative treatment for localized diseases. Highlighted, at diagnosis, metastases frequently uncover in approximately 25% of RCC patients, and the 5-year survival rate among these patients with advanced stage is poor (5–10%) owning to tumor recurrence or distant metastasis [2, 3]. Recently, the improved survival rate of advanced RCC patients has been achieved by a targeted therapy [4, 5]. Nevertheless, nearly all of these patients ultimately relapse or show distant metastasis because of the acquired resistance to targeted therapeutic drugs. Therefore, to identify more molecules responsible for RCC metastasis could offer more novel approaches for developing therapies that can block the RCC metastatic process.

FBXW7 is a member of the F-box family [6, 7], which has been identified to regulate several well-known oncoproteins, such as cyclin E1, c-Myc, Notch, mTOR, and c-Jun, participating in ubiquitin-dependent proteolysis [8]. FBXW7 governs diverse cellular procedures, comprising cell proliferation, differentiation, maintenance of genomic stability, cell-cycle progression, and neural cell stemness [8]. Generally, FBXW7 is regarded as a tumor suppressor in a variety of human cancers. Nevertheless, the function of FBXW7 in tumor metastasis is not commonly reported.

Metastasis is an essential factor correlated with a poor prognosis of RCC patients [1, 9]. Increasing evidence revealed tumor metastasis was correlated with epithelial to mesenchymal transition (EMT) process [10]. EMT involves in profound phenotypic variations that encompass the loss of cell-to-cell adhesion, cell polarity, and the obtainment of migratory and invasive capacities. In addition, the mesenchymal state is believed to be associated with the ability of cells to migrate to distant organs and preserve stemness, permitting their subsequent differentiation into diverse type of cells during the initiation and development of metastasis [10]. Matrix metalloproteinases (MMPs) also play a major role during cancer migration (adhesion and dispersion) and metastasis. MMPs can promote cancer metastasis through degradation of extracellular matrix (ECM) protein [11]. For instance, MMP-2, MMP-9, and MMP-13 belong to MMP superfamily and are markedly upregulated in the stroma during the migration and metastasis processes.

However, rare studies have investigated the mechanisms of metastasis and EMT in RCC. Furthermore, no study has examined the role of FBXW7 in renal cell carcinoma (RCC) metastasis and EMT. With respect to these notions, the present study was performed to investigate the underlying mechanisms of FBXW7 in regulating metastasis and EMT processes in RCC cells.

## 2. Materials and Methods

### 2.1. Cell Lines and Cell Cultures

The human RCC cell lines ACHN, 786-O, and CAKI-1 and normal renal cell line HK-2 were used in our research. These cell lines were purchased from the American Type Culture Collection (ATCC, Rockville, MD, USA). We cultured HK-2 cells in DMEM/F12-MEDIUM (Gibco, Grand Island, NY, USA) and other three cell lines in RPMI-1640-MEDIUM (Gibco). All these mediums were supplied with 10% fetal bovine serum (FBS) (Gibco), as well as 0.8% penicillin/streptomycin (Gibco). We incubated these cell lines at 37°C in an incubator with 5% CO_2_ in a humidified atmosphere.

### 2.2. RNA Extraction and Quantitative Real-Time PCR (qRT-PCR)

We extracted the total RNA by employing TRIzol reagent (Takara Biotechnology Inc., Dalian, China) referring to the manufacturer's instructions. 1-2 *μ*g of total RNA was subjected to reverse transcription using PrimeScript RT reagent Kit (TaKaRa). Quantitative real-time PCR (qRT-PCR) was conducted using an ABI Stepone system with SYBR green to determine the mRNA expression level of a gene of interest. The primer sequences were provided as follows: FBXW7 forward, 5′-AAGGGCAACAACGACG-3′; reverse, 5′-AGGGAGCAATGAAATGAAGT-3′. Twist1 forward, 5′-GGAGTCCGCAGTCTTACGAG-3′; reverse, 5′-TCTGGAGGACCTGGTAGAGG-3′. GAPDH forward, 5′-CTGGGCTACACTGAGCACC-3′; reverse, 5′-AAGTGGTCGTTGAGGGCAATG-3′.

### 2.3. Establishment of Stable FBXW7-Overexpressing Cells

The lentivirus covered coding region FBXW7 cDNA sequence, and the negative control lentivirus was purchased from Genechem (GeneChem Inc., Shanghai, China). To generate overexpressed FBXW7 stable clones, 786-O and ACHN were transfected with lentiviral vectors. The cells were transfected using 5 *μ*g/mL polybrene (GeneChem Inc.) for 24 hours. After that, 10 *μ*g/mL puromycin was used to select the eligible cells. In addition, the efficiency of FBXW7 overexpression was assessed by qRT-PCR and Western blot.

### 2.4. Western Blotting

Cells were lysed with a lysis buffer (Beyotime Biotechnology, Shanghai, China). We employed 10% SDS-PAGE to separate the equal amounts of extracted protein and transferred them onto PVDF membranes. Then, these membranes were probed by first antibody FBXW7 (Rabbit against Human 1 : 1000, Abcam, MA, USA), E-cadherin (Rabbit against Human 1 : 1000, Cell Signaling Technology, MA, USA), N-cadherin (Rabbit against Human 1 : 1000, Abcam), Vimentin (Rabbit against Human 1 : 2000, Abcam), Snail1 (Rabbit against Human 1 : 2000, Proteintech Group Inc., Wuhan, China), MMP-2 (Rabbit against Human 1 : 2000, Proteintech), MMP-9 (Rabbit against Human 1 : 2000, Proteintech), MMP-13 (Rabbit against Human 1 : 2000, Proteintech), and GAPDH (Rabbit against Human 1 : 3000, Abcam) and then incubated with the secondary antibody (Goat against Rabbit 1 : 1000, Goodhere, Hangzhou, China). We used BeyoECL PLUS Kit (Beyotime Biotechnology) to detect the signals referring to the instructions of the manufacturer.

### 2.5. Wound Healing Assay

A sum of 5 × 10^5^ cells were seeded into a six-well plate (Corning, New York, USA). After 24 h incubation, we used sterile plastic 200 *μ*l micropipette tips to wound the monolayer cells. The wounded area pictures were taken by an inverted microscope (Olympus Corp, Tokyo, Japan) immediately (time 0 h) at 24 h. We measured the migration distance of scratches after the photographs were transferred to Photoshop files.

### 2.6. Transwell Assay

The invasion capabilities of RCC cells were determined by the transwell assays. To prepare chambers for assay, 10 mL of Matrigel (BD Biosciences, New Jersey, USA) was dissolved in 50 mL serum-free RPMI-1640, and 100 *μ*l mixed Matrigel was added to the upper chambers of transwells containing 8 *μ*m-pore-size polycarbonate membrane filters (Corning, New York, USA), and put into the incubator for 5 hours. RCC cells were then harvested and seeded with serum-free RPMI-1640 media into the upper chamber at 1 × 10^5^ cells/well, the bottom chambers contained RPMI-1640 with 10% FBS, and then transwells were incubated for 48 hours at 37°C. Following incubation, the invaded cells attached to the lower surface of the membrane were fixed using 4% paraformaldehyde and stained with 1% toluidine blue. Cell numbers were counted in six randomly chosen microscopic fields (100×) per membrane.

### 2.7. Immunofluorescence Analysis

Cells were plated on culture slides (Costar, Manassas, USA). After 24 hours, the cells were rinsed with phosphate buffered saline (PBS) (Gibco) and fixed with 4% paraformaldehyde in PBS, and cell membrane was permeabilized using 0.5% Triton X-100. These cells were then blocked for 30 min in 10% BSA (Sigma-Aldrich, St. Louis, MO, USA), in PBS, and incubated with primary antibodies in 10% BSA overnight at 4°C against Vimentin (Rabbit against Human 1 : 200, Abcam). After three washes in PBS, the slides were incubated for 1 hour in the dark with Alexa Fluor 488-conjugated secondary antibody (Goat against Rabbit 1 : 500, Abcam). After three further washes, the slides were stained with 4-,6-diamidino-2-phenylindole (DAPI; Sigma-Aldrich, St. Louis, MO, USA) for 5 min to visualize the nuclei and examined using an Olympus Corp imaging system (Olympus Corp).

### 2.8. Statistical Analysis

We employed SPSS 19.0 (IBM SPSS, Armonk, NY, USA) to conduct all the statistical analysis. Two-tailed Student's *t*-tests were employed to verify the statistical significance of all these results, and *P* value less than 0.05 was regarded as statistically significant.

## 3. Results

To continue our previously published work [12], we further investigated the function role and underlying mechanisms of FBXW7 in regulating metastasis and EMT processes in RCC cells.

### 3.1. FBXW7 Is Downregulated in RCC Cells Lines

**Figure 1:**
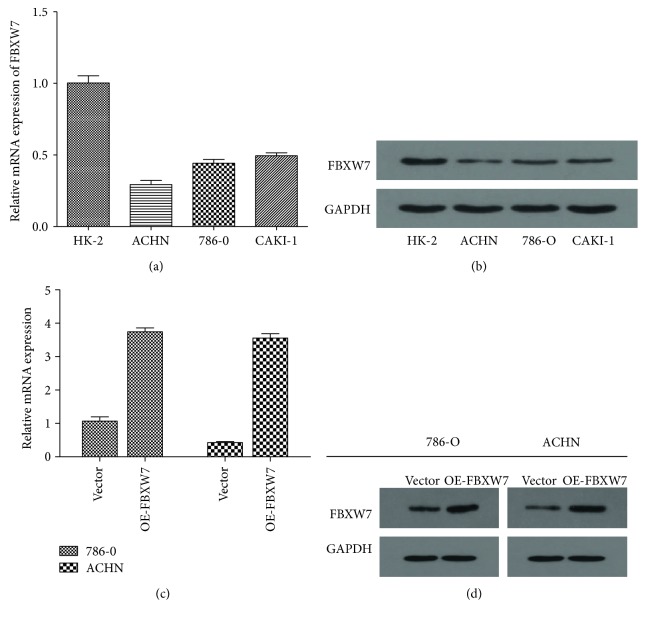
FBXW7 is lowly expressed in three RCC cell lines compared with HK-2 cell line. (a) Expression level of FBXW7 mRNA was detected by qRT-PCR analysis. (b) Expression level of FBXW7 protein was detected by Western blotting analysis. (c) The efficiency of FBXW7 overexpression was examined by qRT-PCR in transfected 786-O and ACHN cell lines. (d) The efficiency of FBXW7 overexpression was examined by Western blotting in transfected 786-O and ACHN cells. All results are from the three independent experiments.

To investigate the FBXW7 expression, qRT-PCR and WB assays were recarried out in RCC cell lines. The results proved that FBXW7 was downregulated in the three RCC cell lines when compared to normal renal cell line HK-2 (Figures 1(a) and 1(b)). In addition, of them, 786-O and ACHN were predominantly downregulated; then, we adopted these two cell lines for further investigations.

### 3.2. Upregulation of FBXW7 Expression Inhibited RCC Cell Migration and Invasion *In Vitro*

**Figure 2:**
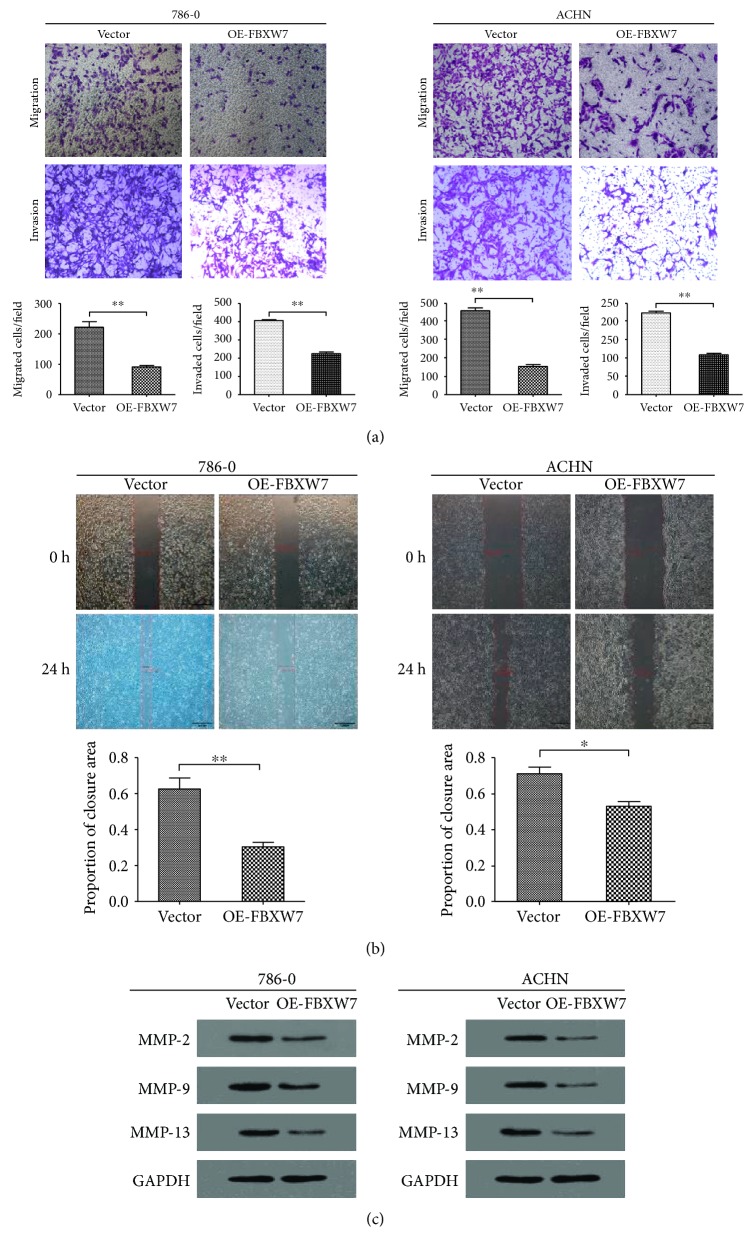
Overexpression of FBXW7 inhibited renal cancer cell migration and invasion. (a) The migration and invasion abilities were analyzed by Boyden chamber assay. (b) The migration was measured by wound healing assay. Scale bars: 50 *μ*m (a) and 500 *μ*m (b). ^∗^*P* < 0.05 and ^∗∗^*P* < 0.01 based on the Student *t*-test. Data are represented as mean ± SD. (c) The expressions of MMP-2, MMP-9, and MMP-13 were measured by Western blotting. GAPDH was probed as the loading control. All results are from the three independent experiments.

As expression of FBXW7 was reported to be positively associated with lymph node and distant organ metastases [13, 14], contributing to the poorer survival, we further explored the effects of FBXW7 overexpression on 786-O and ACHN cells' migration and invasion. Firstly, the cell lines were treated with lentivirus carrying FBXW7 coding sequence and the overexpression efficiency was determined by qPCR and WB. After that, the endogenous FBXW7 protein expression level in 786-O and ACHN cells was efficiently upregulated (Figures 1(c) and 1(d)). Subsequently, to assess the effects of FBXW7 expression on RCC cell migration, a Transwell migration assay was performed using 786-O and ACHN cells. As shown in the figures, fewer FBXW7-transfected cells migrated through the 8 *μ*m pores of the transwell chambers when compared with the control groups (Figure 2(a)). Moreover, similar results were obtained in the invasion assay in which the transwell chambers were precoated with Matrigel (Figure 2(a)). Furthermore, similar results were identified in wound healing assay. Upregulation of FBXW7 had significantly slower closure of the wound area compared to their respective control cells (Figure 2(b)). All these results indicated that upregulation of FBXW7 inhibits the capacity of metastasis in RCC cells.

### 3.3. Upregulation of FBXW7 Decreased MMP-2, MMP9, and MMP-13 in RCC

The degradation of collagenous ECM by MMPs is an essential part for the metastasis of malignant cells [15]. Therefore, we decided to compare the expressions of MMP-2, MMP-9, and MMP-13 in the transfected 786-O and ACHN with control cells and identified that the protein levels of these three proteins were significantly decreased by upregulating the expression of FBXW7 (Figure 2(c)). These results also indicated that FBXW7 suppresses RCC metastasis via decreasing the expressions of MMP-2, MMP-9, and MMP-13.

### 3.4. Upregulation of FBXW7 Inhibited EMT in RCC

**Figure 3:**
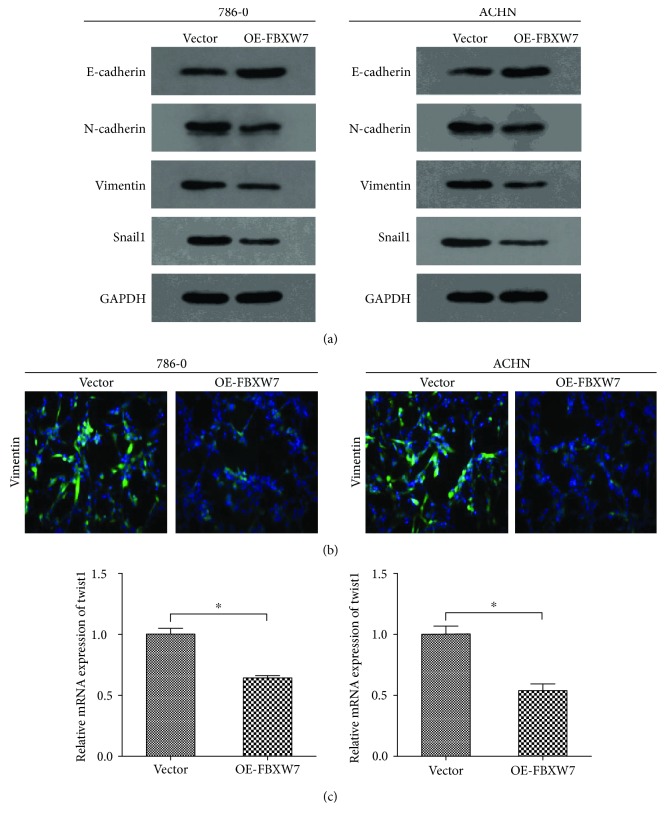
Overexpression of FBXW7 can induce epithelial-mesenchymal transition (EMT). (a) The expression of EMT markers of E-cadherin, N-cadherin, Vimentin, and Snail1 was analyzed by Western blotting. GAPDH was probed as the loading control. (b) Immunofluorescence staining analyzed EMT marker Vimentin (green), and the nuclei were stained with DAPI (blue). (c) mRNA expression level of Twist1 was detected by qRT-PCR analysis. All results are from the three independent experiments. ^∗^*P* < 0.05 based on the Student *t*-test. Data are represented as mean ± SD.

Previous studies have reported that FBXW7 can regulate EMT process in several cancer types [13, 16, 17]. In the current work, we employed WB assay to determine the expression of EMT markers in the transfected 786-O, ACHN, and their control cells. We uncovered that upregulation of FBXW7 inhibited the expression of two classical mesenchymal cell markers (N-cadherin and Vimentin) and Snail1 in both 786-O and ACHN cells, whereas the epithelial cell marker, E-cadherin expression, was elevated, suggesting that a potential connection between FBXW7 and EMT expressions existed (Figure 3(a)). The EMT marker Vimentin was further confirmed by Immunofluorescence analysis (Figure 3(b)). Finally, qRT-PCR studies showed that expression levels of Twist1 was decreased in the transfected cell lines in comparison to the control cells (Figure 3(c)). Combined results suggested that upregulation of FBXW7 suppresses RCC metastatic capacity potentially by inhibiting EMT.

## 4. Discussion

FBXW7, a substrate recognition component of the complex of SKP1, CUL1, and F-box proteins (SCF complex), can bind to its substrates, which have been phosphorylated within conserved phosphodegron motifs, and target them for ubiquitylation and subsequent degradation by the proteasome [18]. Whereas, the functional roles of FBXW7 in metastasis and EMT processes of renal cell carcinoma (RCC) are rarely reported, and the molecular pathways by which FBXW7 regulates RCC metastasis and EMT merit investigation.

In this report, we delineated the mechanistic role of FBXW7 in regulating RCC metastasis. We found that FBXW7 was downregulated in RCC cell lines when compared with normal renal cell HK-2. To evaluate the contribution of FBXW7 to the regulation of RCC, its expression was upregulated in the 786-O and ACHN cell lines, and the subsequent migration and invasion results showed that upregulation of FBXW7 inhibited metastatic capacity of RCC. Furthermore, the protein levels of MMP-2, MMP-9, and MMP-13 indicate that FBXW7 inhibits RCC cell metastatic through MMPs. Cancer invasion is a major part in the process of metastasis, and ECM degradation by MMPs can facilitate the cancer invasion [15]. In addition, several well-known signal pathways are also involved in this procession, including EMT [19]. In the present study, Western blot and Immunofluorescence analyses indicated that upregulation of FBXW7 inhibited EMT. These results indicated that FBXW7 may have a prominent role in the EMT process of RCC. Of course, E-cadherin and N-cadherin are also very important markers of EMT, which are not tested by IF. That is one of the limitations of the current work.

Previously published studies have reported that cancer cells could dedifferentiate to achieve the ability of migration and invasion through triggering the expression of specific genes related to EMT signaling pathway, ensuring cancer cells to spread from the primary tumor site to distant organs. Similarly, Yang et al. [16] identified that FBXW7 suppresses EMT, stemness, and metastatic potential of cholangiocarcinoma cells. Li et al. [17] reported that FBXW7 regulates tumor apoptosis, growth arrest, and the EMT in gastric cancer. Therefore, we predicted that upregulation of FBXW7 suppresses RCC cell metastasis also through EMT pathway. These observations above confirmed the crucial role of FBXW7 in RCC, and further studies will be continued on this issue.

In summary, the present study is the first to report that FBXW7 suppressed RCC metastatic capacity. Furthermore, FBXW7 suppresses RCC metastasis capacity potentially through EMT process. However, the molecular mechanisms by how FBXW7 can regulate the RCC migration and invasion require further investigation. Understanding the critical role of FBXW7 in RCC may lead to the development of a novel diagnostic marker for this type of cancer.
